# Hydrogen sulphide decreases IL-1β-induced activation of fibroblast-like synoviocytes from patients with osteoarthritis

**DOI:** 10.1111/jcmm.12405

**Published:** 2014-10-14

**Authors:** Daniela Sieghart, Melissa Liszt, Axel Wanivenhaus, Hans Bröll, Hans Kiener, Burkhard Klösch, Günter Steiner

**Affiliations:** a Division of Rheumatology, Department of Internal Medicine III, Medical University of ViennaVienna, Austria; b Ludwig Boltzmann Cluster for Rheumatology, Balneology and Rehabilitation, Institute for Rheumatology and BalneologyVienna-Oberlaa, Austria; c Department of Orthopaedics, Medical University of ViennaVienna, Austria

**Keywords:** hydrogen sulphide, fibroblast-like synoviocytes, inflammation, signal transduction

## Abstract

Balneotherapy employing sulphurous thermal water is still applied to patients suffering from diseases of musculoskeletal system like osteoarthritis (OA) but evidence for its clinical effectiveness is scarce. Since the gasotransmitter hydrogen sulphide (H_2_S) seems to affect cells involved in degenerative joint diseases, it was the objective of this study to investigate the effects of exogenous H_2_S on fibroblast-like synoviocytes (FLS), which are key players in OA pathogenesis being capable of producing pro-inflammatory cytokines and matrix degrading enzymes. To address this issue primary FLS derived from OA patients were stimulated with IL-1β and treated with the H_2_S donor NaHS. Cellular responses were analysed by ELISA, quantitative real-time PCR, phospho-MAPkinase array and Western blotting. Treatment-induced effects on cellular structure and synovial architecture were investigated in three-dimensional extracellular matrix micromasses. NaHS treatment reduced both spontaneous and IL-1β-induced secretion of IL-6, IL-8 and RANTES in different experimental settings. In addition, NaHS treatment reduced the expression of matrix metallo-proteinases MMP-2 and MMP-14. IL-1β induced the phosphorylation of several MAPkinases. NaHS treatment partially reduced IL-1β-induced activation of several MAPK whereas it increased phosphorylation of pro-survival factor Akt1/2. When cultured in spherical micromasses, FLS intentionally established a synovial lining layer-like structure; stimulation with IL-1β altered the architecture of micromasses leading to hyperplasia of the lining layer which was completely inhibited by concomitant exposure to NaHS. These data suggest that H_2_S partially antagonizes IL-1β stimulation *via* selective manipulation of the MAPkinase and the PI3K/Akt pathways which may encourage development of novel drugs for treatment of OA.

## Introduction

Osteoarthritis (OA) is a progressive disease of the joints that is characterized by an imbalance of synthesis *versus* degradation of extracellular matrix tissue. The secretion of pro-inflammatory cytokines, predominantly interleukin (IL)-1β, is among the critical steps mediating the aberrant degenerative processes seen in OA pathophysiology in which fibroblast-like synoviocytes (FLS) play a pivotal role [1–3]. FLS are localized in the intimal lining layer of the synovial membrane which encapsulates the synovial joint, and are responsible for the expression of UPD-glucose 6-dehydrogenase, which is required for the synthesis of hyaluronic acid and CD55 (decay accelerating factor) [4]. Furthermore, synovial fibroblasts secrete extracellular matrix components such as collagens and proteoglycans, for example lubricin [5]. Upon stimulation FLS are capable of producing large amounts of IL-6 and IL-8, both of which are key players in inflammatory diseases, and also the chemokine regulated on activation, normal T cell expressed and secreted (RANTES), acting as a chemoattractant and belonging to the IL-8 superfamily. In addition, FLS produce extracellular matrix degradative enzymes like matrix metallo-proteinases (MMPs) which have been found to be overactivated in several pathologic conditions, including OA [6].

Although OA is the most prevalent of the rheumatic diseases and a major cause for morbidity amongst the elderly [7], therapies efficiently inhibiting disease progression are not available yet. Sulphur baths that are rich in hydrogen sulphide (H_2_S) are therefore still considered a therapeutic option and balneotherapy has been included in the recently published OARSI guidelines for treatment of OA of the knee [8]. However, strong evidence for clinical effectiveness is scarce and the molecular basis for the reported beneficial effects is poorly understood [9–12]. Hence, the underlying molecular pathways need to be thoroughly investigated to further the development of novel sulphur releasing drugs that could ameliorate this condition [13].

Hydrogen sulphide is a member of the gasotransmitter family showing multiple physiological effects and is endogenously produced from L-cysteine by the enzymes cystathionine-β-synthase and cystathionine-γ-lyase [14]. H_2_S plays an important role in modulating inflammation by affecting several key mechanisms and pathways including (*i*) suppression of leucocyte adherence and migration, (*ii*) reduction in expression of pro-inflammatory mediators, (*iii*) promotion of vasodilation and angiogenesis as well as reduction in NF-κB activation [15]. Exogenous H_2_S sources for example are sodium hydrogen sulphide (NaHS), sodium sulphide (Na_2_S) or synthetic compounds like GYY4137 [16]. NaHS is a sulphur salt which acts as exogenous H_2_S donor when in solution and is therefore frequently used for studying the effects of H_2_S *in vitro* [15,17]. Previous studies performed by us and other investigators suggest controversial effects of NaHS treatment which, dependent on the experimental conditions, may show both anti- and pro-inflammatory effects [18–22]. To gain more insight into the biological activity of H_2_S, we explored the effects of NaHS treatment on IL-1β-activated synovial fibroblasts derived from OA patients. The data obtained strongly suggest that H_2_S has pronounced anti-inflammatory effects *in vitro* that might interfere with the activation of several signal transduction pathways.

## Materials and methods

### Antibodies and reagents

If not stated otherwise, reagents were obtained from Sigma-Aldrich (Vienna, Austria) and Carl Roth GmbH (Karlsruhe, Germany). Oligonucleotides for qRT-PCR were purchased from Eurofins-MWG Operon (Ebersberg, Germany). Antibodies specific for p-MKK3/6, p-Akt, p-GSK-3β or β-actin were purchased from Cell Signaling Technology (Danvers, MA, USA).

### Specimen collection and cell culture

Primary FLS used in this study had been obtained previously upon synovectomy from synovial tissue of seven OA patients and were stored in liquid nitrogen [18,23]. After thawing, FLS were cultured until confluency as described [18] and used between passage four and 14. In most experiments cells were stimulated with 10 ng/ml of IL-1β (Prospec, Ness Ziona, Israel). If not stated otherwise, cells were treated with 1 mmol/l NaHS freshly prepared in PBS and diluted appropriately.

### Proteome profiler array

Phosphorylation of 26 different MAPkinases or other serine/threonine kinases was analysed by spot blot assay (R&D Systems Inc., Minneapolis, MN, USA). FLS from three different patients were exposed to four different treatments (PBS, IL-1β, IL-1β + NaHS, NaHS) and cell lysates were prepared according to the manufacturer's instructions. Afterwards, protein concentrations were measured by Bradford assay and properly diluted samples were mixed with 20 μl of reconstituted detection antibody cocktail and incubated for 1 hr. A total of 12 nitrocellulose membranes were then probed overnight at 4°C and subsequently incubated with horseradish peroxidase-linked Streptavidin. Proteins were visualized by chemiluminescence using the imaging system ChemiDoc XRS or on photographic film after an exposure of 8 min. Pixel densities for each kinase blotted in duplicates were quantified using the imaging system GeneGnome (Syngene, Cambridge, UK).

### Western blotting

Cells were lysed in sample buffer (R&D Systems Inc.) for 30 min on a rocking plate at 4°C following which they were centrifuged. Resulting lysates were mixed with Laemmli sample buffer (Bio-Rad Laboratories, Hercules, CA, USA) supplemented with β-Mercaptoethanol and incubated for 5 min. at 95°C. After separation on a 7.5% polyacrylamide-SDS gel, proteins were transferred to a 0.2 μm nitrocellulose membrane by electroblotting. The membrane was then blocked for 1 hr in washing solution (PBS supplemented with 0.1% Tween 20 and 5% dry milk). Subsequently, the blots were probed overnight at 4°C with the primary antibody. After washing, the membrane was incubated for 1 hr with the appropriate secondary antibody. Results were visualized by chemiluminescence detection on photographic films.

### Micromass culture

Three-dimensional (3-D) cultures were constructed as described previously [5,24]. Briefly, cultured FLS were released from the culture dish, counted and pelleted. The pellet was resuspended in ice-cold Matrigel Matrix (BD Biosciences, San Jose, CA, USA) at a density of 5 × 10^6^ cells/ml. The solution was transferred one drop each to the middle of a poly-2-hydroxyethylmethacrylate (poly-HEMA) pre-coated culture well. Afterwards, micromass culture medium composed of standard medium supplemented with 1% ITS liquid media supplement (BD Biosciences), bovine serum albumin (1.250 g/l) and vitamin C (0.176 g/l) was added to each well. The medium was changed three times a week. The floating micromasses were treated and incubated with IL-1β and/or NaHS as described in the results section. FLS from three patients were used to create a total of 27 micromass cultures—nine cultures per patient performed in triplicates—which were then treated with either PBS (*n* = 9), IL-1β (*n* = 9) or IL-1β + NaHS (*n* = 9) on day 3, 7, 10, 13 and 15. Micromasses were fixed with 2% paraformaldehyde in PBS. After dehydration and paraffin-embedding the cultures were sectioned at 4 μm thickness and haematoxylin and eosin staining was performed. For quantification of the lining layer-like area Osteomeasure software (OsteoMetrics Inc., Decatur, GA, USA) was used. Lining thickness was calculated in μm^2^ allowing quantitative comparison of the different treatments applied.

### Immunohistochemistry

Slides were deparaffinized, rehydrated and subjected to antigen retrieval. After blocking unspecific binding sites for 10 min., the primary antibody specific for p-Akt or MMP-2 diluted in goat serum was applied and incubated for 1 hr. Following 30 min. incubation with the secondary antibody, the Vectastain avidin-biotin complex (Vector laboratories, Peterborough, UK) was added for 45 min. and colour was developed using 3,3′-diaminobenzidine tetrahydrochloride. Slides were counterstained with haemalaun to visualize the nucleus. Light microscopy images were captured and images were processed using Adobe Photoshop CS6 software (Adobe Systems, San Jose, CA, USA).

### Flow cytometric analysis

Following treatment, FLS were washed with labelling buffer (10 mmol//l Hepes/NaOH, pH 7.4, 140 mmol/l NaCl, 2.5 mmol/l CaCl_2_, filtered) and labelled with annexin V (eBioscience, Vienna, Austria). Afterwards, cells were stained with labelling buffer containing 0.001 g/l 7-Aminoactinomycin (7-AAD) and processed in flow cytometry (BD Biosciences Facscanto II, with Facsdiva software). For induction of apoptosis, FLS were treated for 10 min. with 1 joule (J)/cm^2^ UV at 254 nm.

### Quantification of cytokines/chemokines

The amount of secreted IL-6, IL-8 and RANTES was measured out of cell culture supernatants by ELISA obtained from eBioscience or R&D Systems Inc. according to the manufacturer's specifications.

### qRT-PCR

Total RNA was isolated from FLS using TriFast (Peqlab, Erlangen, Germany) and reverse transcribed with random hexamers (Finnzymes, Espoo, Finland). qRT-PCR was performed with a DyNAmo SYBR Green qPCR kit (Finnzymes) under the following thermal conditions: 95°C for 7 min. and 40 cycles of 95°C for 20 sec., 60°C for 30 sec. and 72°C for 15 sec. GAPDH was used as a normalizing control. The gene sequence of GAPDH (NM_002046.3) was derived from Ensembl Human Genome Browser. Forward (5′-CGG GGC TCT CCA GAA CAT C-3′) and reverse (5′-CTC CGA CGC CTG CTT CAC-3′) oligonucleotide primers were designed using the primer three programme and purchased from Eurofins-MWG Operon (Ebersberg, Germany). Oligonucleotide primers specific for MMP-2 (forward: 5′-GCG ACA AGA AGT ATG GCT TC-3′ and reverse: 5′-TGC CAA GGT CAA TGT CAG GA-3′) and MMP-14 (forward: 5′-CAA CAC TGC CTA CGA GAG GA-3′ and reverse: 5′-GTT CTA CCT TCA GCT TCT GG-3′) were derived from Konttinen *et al*. [25].

### Statistical analysis

All statistic evaluations were performed with GraphPad Prism (Texas Instruments, La Jolla, CA, USA) version 5. Data are expressed as the mean ± SEM. Homogeneous distribution of variances was analysed by *f*-tests. For normally distributed data, an unpaired Student's *t*-test with Welch's correction was used. In case of heterogeneous variances, a log transformation of data sets was applied prior to analysis. *P*-values less than 0.05 (*), 0.01 (**) or 0.001 (***) were considered statistically significant.

## Results

### NaHS inhibits spontaneous IL-6 production in FLS

**Fig. 1:**
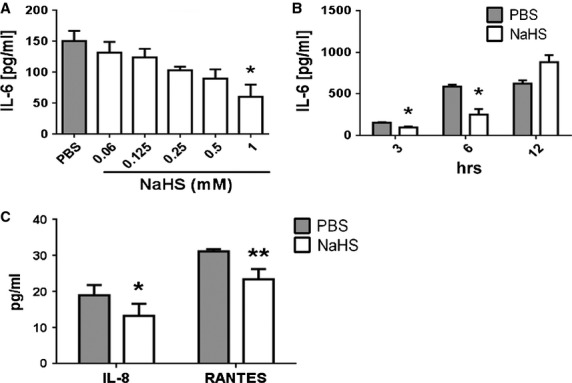
Sulphur treatment decreases IL-6 secretion in resting FLS. (**A**) FLS were treated with increasing concentrations of NaHS (0.06–1 mmol/l) for 1 hr and after 1 hr recovery in sulphur-free medium, IL-6 production was measured in cell culture supernatants by ELISA. (**B**) Cells were treated with PBS or 1 mmol/l NaHS for 1 hr following which they were allowed to recover in fresh medium for 3, 6 or 12 hrs. Supernatants were analysed for IL-6 concentration by ELISA. (**C**) Supernatants from (**A**) were further used to quantify human IL-8 and RANTES of FLS treated with 1 mmol/l NaHS or PBS. FLS from four different OA patients were used in these experiments. Each experiment was performed three times in duplicates or two times in triplicates (**A**) *P < 0.05; (**B**) *P < 0.05; (**C**) *P < 0.05, **P < 0.01.

Data obtained previously with a FLS line from a patient with OA had shown stimulatory effects of a 20 min. short-term exposure to H_2_S [19]. To investigate whether prolonged exposure to H_2_S could inhibit spontaneous cytokine production, FLS derived from seven OA patients (OA-FLS) were incubated for 1 hr with increasing concentrations (0.06–1 mmol/l) of the H_2_S donor NaHS or PBS as negative control; then the incubation medium was replaced with fresh medium and incubation continued for an additional hour. Although OA-FLS under resting conditions have been reported to produce no or only small amounts of IL-6 [3,26], among the seven primary FLS cultures investigated four were found to spontaneously secrete moderate amounts of IL-6. In all four cultures sulphur treatment led to the reduction in spontaneous IL-6 secretion in a concentration-dependent manner (Fig. 1A) and in the remaining three cultures stimulation of IL-6 production was not observed (not shown). Maximum inhibition (∼60%) was observed at 1 mmol/l NaHS concentration which was therefore used in all subsequent experiments.

To elucidate the persistence of this inhibitory effect, FLS were exposed to 1 mmol/l NaHS for 1 hr, and IL-6 was quantified following 3, 6 and 12 hrs recovery in sulphur-free medium. IL-6 secretion was significantly decreased in supernatants of NaHS-treated FLS following 3 and 6 hrs recovery (Fig. 1B) while an inhibitory effect was no longer observed after 12 hrs. To investigate potential effects on chemokine secretion the chemokines IL-8 and RANTES were quantified in addition. Under resting conditions all four FLS cultures investigated secreted low amounts of both chemokines and 1 hr treatment with 1 mmol/l NaHS followed by 1 hr recovery in fresh medium significantly decreased secretion of RANTES and IL-8 by ∼25% (Fig. 1C).

### NaHS does not affect viability of FLS

**Fig. 2:**
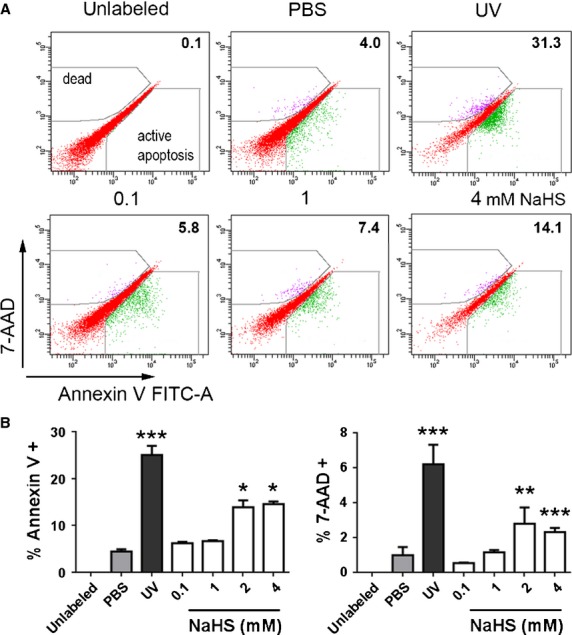
NaHS does not affect viability of FLS at therapeutically relevant concentrations. Viability of FLS was determined by flow cytometry employing annexin V staining, following 1 hr treatment with different concentrations of NaHS. For a negative control, cells were incubated with PBS. For a positive control, cell death was induced by 10 min. UV irradiation at 254 nm. (**A**) FACS scatter plots of one of three independent experiments: the percentages of early apoptotic cells are indicated. (**B**) Quantitative evaluation of three independent experiments employing FLS from two different OA patients tested in duplicates. (**B**) *P < 0.05, ***P < 0.001.

Since H_2_S is toxic at high concentrations [14], we wanted to investigate the susceptibility of FLS to H_2_S toxicity. Cells from two OA patients were treated for 1 hr with increasing concentrations of NaHS or were irradiated with UV light as positive control. After 24 hrs recovery, cellular viability was determined by flow cytometry of annexin V stained cells. At NaHS concentrations up to 1 mmol/l no significant effect on cellular viability was observed (Fig. 2A and B). Cellular viability decreased significantly at concentrations of 2 mmol/l and higher (*P* < 0.05). Hence, at concentrations used throughout this study, the physiological effects observed were not because of cytotoxicity.

### NaHS inhibits IL-1β-induced FLS activation

**Fig. 3:**
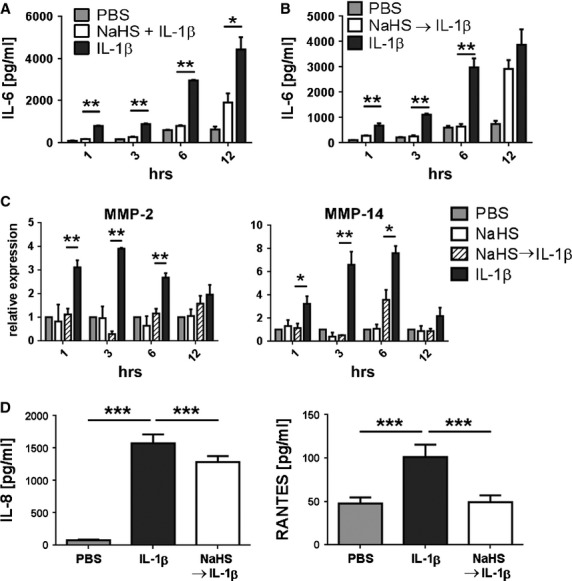
NaHS decreases secretion of IL-6, IL-8 and RANTES and diminishes expression of MMP-2 and MMP-14 in IL-1β -stimulated FLS. FLS were either (**A**) treated simultaneously with IL-1β + NaHS or (**B** and **C**) were pre-treated with NaHS for 1 hr following 1 hr stimulation with IL-1β. PBS and IL-1β-treated FLS were used as negative and positive control, respectively. Cells were further incubated in sulphur-free medium for 1, 3, 6 or 12 hrs. (**A** and **B**) IL-6 secretion was analysed in cell culture supernatants by ELISA; (**C**) cells were harvested and the expression of MMP-2 and MMP-14 was determined by qRT-PCR. Data were normalised to GAPDH expression. (**D**) For measuring IL-8 and RANTES secretion cells were pre-treated with NaHS or PBS for 1 hr, stimulated with IL-1β for an additional h and incubated in fresh medium for 3 hrs. In all experiments FLS from three different patients were tested three times in duplicates or two times in triplicates. (**A**) *P < 0.05, **P < 0.01; (**B**) **P < 0.01; (**C**) *P < 0.05, **P < 0.01 (**D**) ***P < 0.001.

Under pathological conditions FLS may become activated by pro-inflammatory cytokines such as IL-1β, leading to production of large amounts of IL-6 [3,26]. Therefore, it was important to determine whether NaHS affects the secretion of IL-6 also in IL-1β-activated FLS. Cells from four different OA patients were treated simultaneously with 1 mmol/l NaHS and IL-1β for 1 hr. Afterwards, cells were transferred to fresh medium and further incubated for up to 12 hrs. Aliquots of the supernatants were obtained at 1, 3, 6 and 12 hrs and were analysed by ELISA. Following 1 hr recovery in sulphur-free medium, supernatants of PBS-treated control cells contained ∼75 ± 6.5 pg/ml IL-6, whereas in supernatants of IL-1β-activated FLS substantially higher amounts were measured (838 ± 61 pg/ml). In cells treated with NaHS, IL-6 secretion was significantly reduced to 160 ± 34 pg/ml (Fig. 3A) and this inhibitory effect could still be seen after recovery times of 3 and 6 hrs (p < 0.01) and, though less pronounced, even after 12 hrs (p < 0.05). A similar result was obtained when cells were pre-treated with 1 mmol/l NaHS for 1 hr and subsequently stimulated with IL-1β for one additional hour (Fig. 3B). However, when cells were treated with NaHS after 1 hr IL-1β stimulation no inhibitory effects were observed (data not shown).

Overexpression of MMPs is a hallmark of synovial fibroblasts [27]. Since sulphurylated compounds like chondroitin sulphate were found to affect FLS by reducing the expression of MMPs [28], we investigated the ability of NaHS to interfere with tissue remodelling by down-regulating MMP expression. FLS from three OA patients were treated with 1 mmol/l NaHS or PBS for 1 hr, following activation with IL-1β for an additional hour, and allowed to recover in fresh medium for 1, 3, 6 and 12 hrs. Expression of MMP-2 and MMP-14 was analysed by qRT-PCR because MMP-14 harbours a transmembrane domain and is therefore more likely bound to the cell membrane rather than secreted. Moreover, most MMPs are synthesized as inactive precursors which need to be activated by proteolytic cleavage prior to secretion and therefore their concentration in culture supernatants may not always reflect gene activation. After 1 hr recovery an approximately twofold reduction in the expression of MMP-2 and MMP-14 was observed in NaHS-treated samples compared to the PBS controls (Fig. 3C). Longer recovery times (up to 6 hrs) showed up to fourfold (MMP-2) and sixfold (MMP-14) decreased expression in NaHS-treated samples. However, after 12 hrs recovery this inhibitory effect was no longer significant. Hence, the deactivating effects of H_2_S on IL-1β-stimulated MMP expression were transient.

In addition, the effects of NaHS treatment on IL-1β-induced secretion of IL-8 and RANTES was analysed in cell culture supernatants from FLS treated with 1 mmol/l NaHS or PBS for 1 hr prior to a 1 hr activation with IL-1β followed by 3 hrs recovery in fresh medium. IL-1β stimulation induced massive secretion of IL-8 and, although to a lesser extent of RANTES (Fig. 3D). Pre-treatment with NaHS significantly reduced the secretion of IL-8 by ∼20% while RANTES production was decreased to almost baseline levels (Fig. 3D), confirming the anti-inflammatory potential of long-term NaHS treatment.

### NaHS reduces IL-1β-induced MAPkinase activation

Members of the mitogen-activated protein (MAP) kinase family are key players in inflammatory responses. Since expression of IL-6 and several MMPs partially depends on the activation of the MAPkinase pathway [26], we were interested to investigate the effects of NaHS on IL-1β-induced activation of MAPkinases and other serine/threonine kinases. FLS from three different patients were treated for 30 min. with IL-1β, 1 mmol/l NaHS, 1 mmol/l NaHS + IL-1β or PBS as a negative control.

**Fig. 4:**
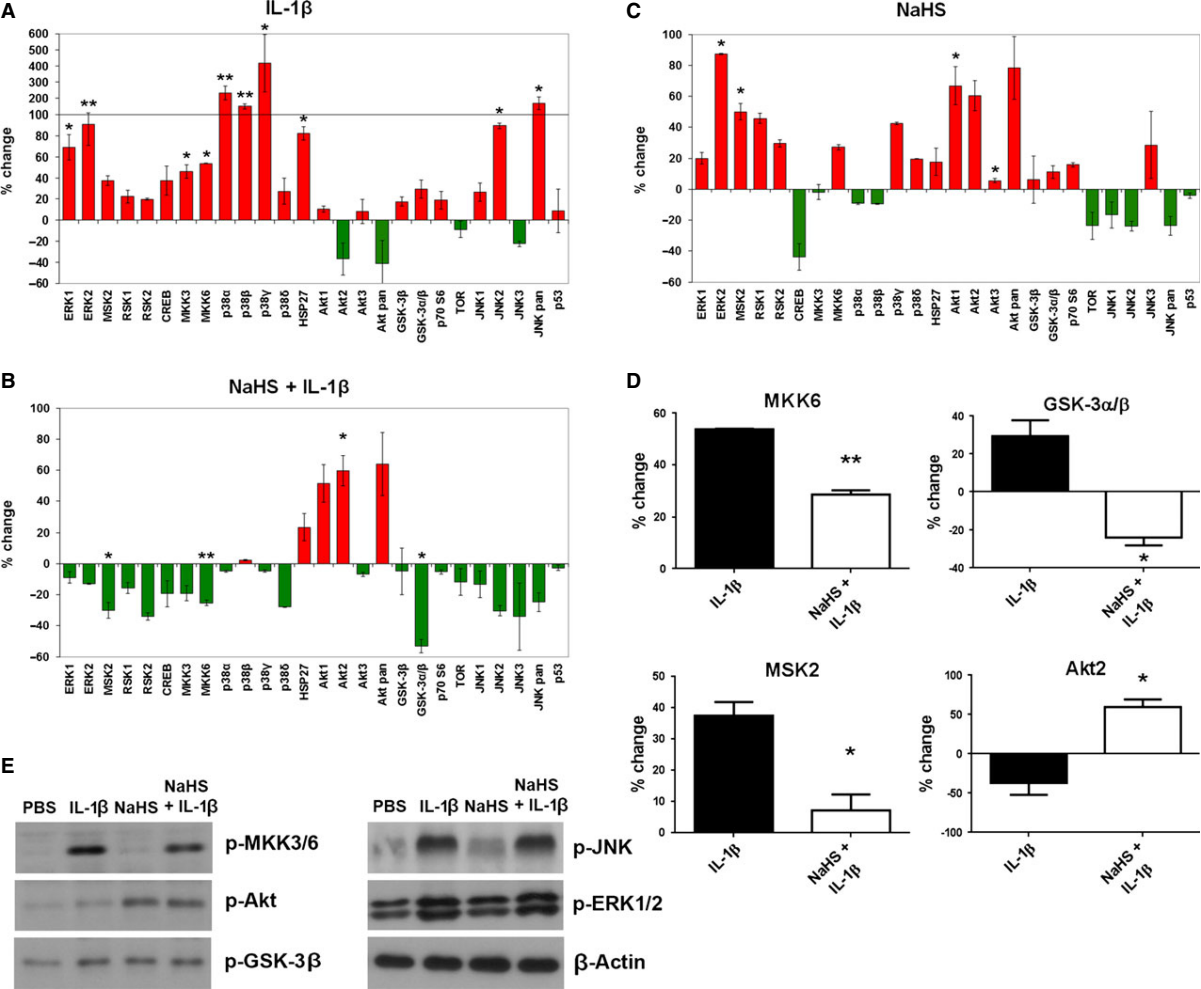
NaHS affects IL-1β-induced phosphorylation of MAPkinases. Phosphorylation of 26 different MAPkinases and other serine/threonine kinases was analysed by spot blot assay. FLS from three different patients were incubated with PBS, IL-1β or NaHS + IL-1β for 30 min. Changes in pixel density were quantified using the imaging system GeneGnome, statistically evaluated and plotted into graphs (% change). (**A**) Changes of phosphorylation in IL-1β-activated FLS (normalised to PBS treated samples) showing significantly increased phosphorylation of multiple kinases, but decreased levels of p-Akt2. (**B**) Changes of phosphorylation in NaHS treated activated FLS (normalised to IL-1β-stimulated samples) showing decreased phosphorylation of multiple kinases but highly increased levels of p-Akt. (**C**) Changes of phosphorylation in unstimulated, NaHS treated FLS showing activation of several kinases and decreased the phosphorylation of CREB and JNK. (**D**) Significant differences in the phosphorylation of MKK6, GSK-3α/β, MSK2 and Akt2 induced by NaHS treatment in IL-1β-activated FLS, normalised to untreated controls. (**E**) Western blot analysis of p-MKK3/6, p-Akt, p-GSK-3β, p-JNK and p-ERK1/2. β-Actin was used as a loading control. (**A**) *P < 0.05, **P < 0.01; (**B**) *P < 0.05, **P < 0.01; (**C**) *P < 0.05, **P < 0.01.

The analysis revealed that IL-1β induced in a statistically significant manner the phosphorylation of several MAPkinases (Fig. 4A) including extracellular signal-regulated kinase (ERK)1/2 (68 ± 16% and 90 ± 27%), mitogen- and stress activated protein kinase (MSK)2 (37 ± 5%), mitogen-activated protein kinase kinase (MKK)3/6 (46 ± 8% and 53 ± 3%), heat shock protein (HSP)27 (82 ± 15%), p38 subunits α (232 ± 51%), β (125 ± 38%) and γ (417 ± 216%) as well as c-Jun-N-terminal kinase (JNK)/2 (89 ± 3%). In contrast, protein kinase B (Akt2) phosphorylation appeared to be slightly down-regulated by IL-1β (−37 ± 26%). Treatment with NaHS reduced the IL-1β-induced phosphorylation of multiple kinases but, interestingly, did not affect the highly activated p38 MAPkinases (Fig. 4B). However, it must be noted that, presumably because of the low sample size, these changes reached the level of statistical significance only for MSK2, MKK6 and GSK3α/β (Fig. 4D). Surprisingly, NaHS increased phosphorylation of Akt1/2 by 50–60% (Fig. 4B and D), an effect that was also observed in unstimulated NaHS-treated cells in which, in line with previous observations [19], activation of ERK2 and its downstream target MSK2 was also seen (Fig. 4C). To further substantiate these findings, p-MKK3/6, p-GSK-3β, p-Akt, p-JNK and p-ERK1/2 were in addition analysed by Western blotting which largely confirmed the data obtained by array analysis (Fig. 4E).

### NaHS treatment abolishes the IL-1β-induced architectural changes of FLS grown in 3-D extracellular matrix micromass cultures

To investigate the effects of NaHS on the cellular architecture within the synovium, FLS were cultured in 3-D micromasses using a preformed matrix. FLS derived from rheumatoid arthritis (RA) patients, grown in micromass cultures, have been described previously to spontaneously form a complex synovial lining layer-like architecture which—following stimulation with TNF-α—displayed lining hyperplasia, characteristic of the hyperplastic rheumatoid synovium [24].

**Fig. 5:**
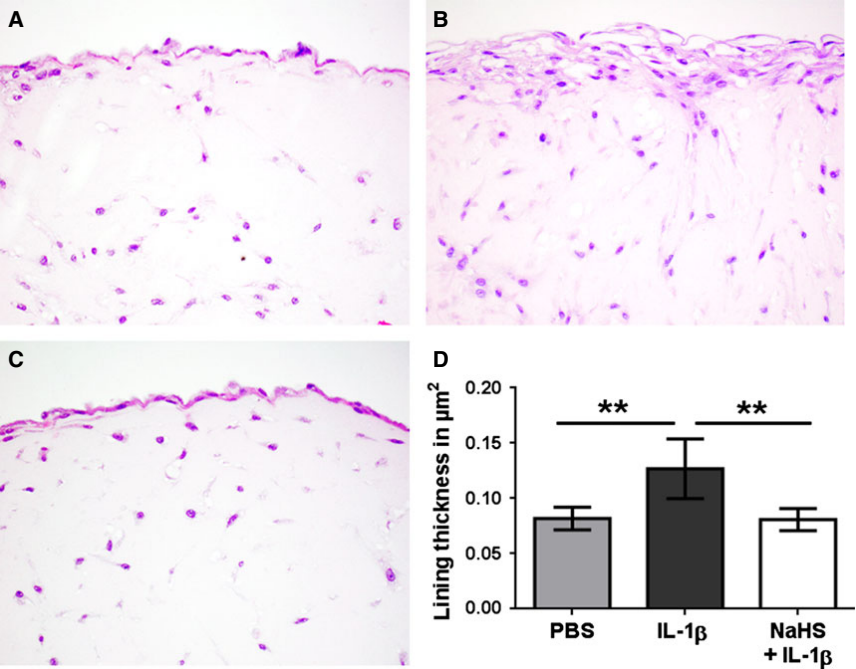
FLS grown in 3-D micromass organ cultures can be activated by IL-1β and are susceptible to NaHS treatment. FLS from 3 patients were used to create a total of 27 micromass cultures—9 cultures per patient performed in triplicates—which were then treated with either PBS (*n* = 9), IL-1β (*n* = 9) or IL-1β + NaHS (*n* = 9) on day 3, 7, 10, 13 and 15. After 17 days, 3-D cultures were fixed, sectioned and stained with haematoxylin and eosin. Representative cultures of one patient treated with (**A**) PBS, (**B**) IL-1β or (**C**) NaHS + IL-1β are shown. Original magnification ×1000. (**D**) Quantification of lining layer thickness in 27 micromasses using Osteomeasure software. (**D**) **P < 0.01.

Micromasses from three different OA patients were treated with either PBS, or IL-1β or IL-1β + 1 mmol/l NaHS on day 3, 7, 10, 13 and 15. The experiment was performed in triplicates and was terminated at day 17 by fixation of micromasses. Quantification of lining layer-like area was performed and representative pictures were made. FLS spontaneously formed a tightly compacted multicellular organization, building a synovial lining-like structure (Fig. 5). IL-1β stimulation led to hyperplasia of the lining layer (Fig. 5B and D). Remarkably, these IL-1β-induced alterations could be completely inhibited by treatment with NaHS (Fig. 5C and D). Thus, treatment with NaHS antagonized the effects of IL-1β on the architecture of micromass cultures.

**Fig. 6:**
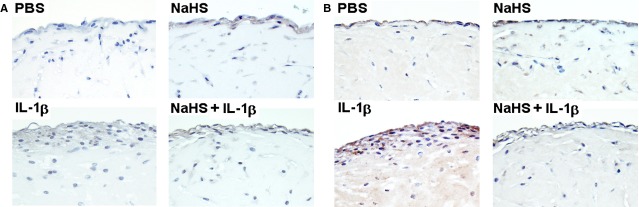
NaHS induces Akt activation and inhibits IL-1β-induced MMP-2 production in 3-D micromass organ cultures. FLS grown in micromass cultures were treated either with PBS, NaHS, IL-1β or a combination of NaHS and IL-1β (see Fig. 5) and were immunhistochemically stained for (**A**) p-Akt or (**B**) MMP-2. Original magnification ×1000.

Immunohistochemical stainings for p-Akt revealed that NaHS treatment increased the activation of Akt in lining cells (Fig. 6A) confirming the data obtained in conventional cultures. Treatment with IL-1β or a combination of NaHS and IL-1β also showed slightly increased levels of Akt activation (Fig. 6A). Additional stainings were performed for MMP-2 which showed a pronounced increase after IL-1β stimulation (Fig. 6B). In cultures treated with NaHS + IL-1β expression of MMP-2 was decreased (Fig. 6B). NaHS treatment alone did not seem to alter MMP-2 production compared to PBS-treated micromasses.

## Discussion

There is growing evidence that H_2_S is an important signalling molecule similar to other gasotransmitters like carbon oxide (CO) or nitric oxide. Its role in inflammation is very complex with both pro- and anti-inflammatory effects described [19,20,22,23,29,30]. In a previous study, we found that short time exposure (20 min.) led to increased IL-6 secretion in unstimulated FLS [19] whereas 1 hr NaHS treatment decreased spontaneous IL-6 synthesis in FLS derived from RA patients [18], suggesting a time dependent effect.

To substantiate these preliminary findings, we have now focused our investigations on FLS from OA patients which, in contrast to FLS from RA patients, do not produce excessive amounts of IL-6 (and other inflammatory mediators), unless stimulated. In a first series of experiments, we found that 1 hr treatment with NaHS significantly reduced in a dose-dependent manner the amounts of spontaneously secreted IL-6 confirming data previously obtained with FLS from RA patients [18]. The inhibitory effect was transient and no longer observed after 12 hrs supporting the observation that H_2_S was not toxic at the concentrations applied. Pronounced inhibition was observed at a concentration of 1 mmol/l which was therefore used in most subsequent experiments. Although this may appear unphysiologically high, it must be taken into consideration that the half-life of H_2_S in aqueous solution is short [31] and that NaHS at this concentration was not toxic for FLS. Furthermore, although measurement of H_2_S levels in biological specimen is technically difficult [32], concentrations of more than 100 μmol/l have been detected in synovial fluid of patients with RA [33]. Of note, sulphur concentration in thermal waters used for therapy of rheumatic and other diseases usually ranges between 0.1 and 0.2 mmol/l [34] but in some springs may even exceed 1 mmol/l, *e.g*. in the spa Vienna-Oberlaa.

To more closely mimic the pathophysiological conditions of FLS in an osteoarthritic joint, we employed IL-1β as an inflammation inducing agent that is pivotally involved in joint inflammation and cartilage destruction [29] and induces excessive production of IL-6 [30]. The efficacy of NaHS in this pro-inflammatory setting was confirmed by its ability to efficiently inhibit IL-1β-induced IL-6 production. These results were further substantiated by analysing other cytokines/chemokines that are important during inflammatory responses such as IL-8 and RANTES both of which were significantly down-regulated by NaHS treatment. The effect was particularly prominent for RANTES whose IL-1β-induced secretion was largely abolished by NaHS.

Another crucial step in the progression of OA is the breakdown of matrix proteins induced by aberrant expression of several MMPs. MMP-2 is a mediator of basement-membrane degradation and, besides several types of collagens, degrades precursors of TNF-α and IL-1β [35]. MMP-2 is activated by binding membrane-anchored MMP-14—both expressed on synovial fibroblasts [36]—and forms a membrane-bound complex with tissue inhibitors of metallo-proteinase-2 [37], which shows matrix degrading potential [36,38]. The observed inhibitory effects of NaHS on the expression of these two MMPs encouraged us to further elucidate the molecular mechanisms by investigating the potential effects of sulphur treatment on signal transduction pathways known to regulate expression and activation of cytokines and MMPs [39–44].

Hydrogen sulphide has already been shown in previous investigations to interfere with MAPkinases [18–20] and the expression of IL-6 [26] and some MMPs [37,41,45] partially depend on their activation. In line with this, NaHS reduced the IL-1β-induced activation of several MAPkinases, particularly MSK2 which acts downstream of p38 and ERK1/2 as well as MKK6 acting upstream of p38 and GSK-3 which is involved in numerous cellular pathways regulating immune and migratory processes, among which deactivation of β-catenin and thus interference with the Wnt signalling pathway may be of particular importance for FLS.

Interestingly, upon IL-1β stimulation a slight (though not significant) reduction in Akt2 phosphorylation was observed. Remarkably, treatment with NaHS did not only revert this effect but also augmented phosphorylation of both Akt1 and Akt2 substantially. The protein kinase B, encoded by the genes Akt1-3 is part of the PI3K/Akt pathway regulating a variety of cellular functions *e.g*. survival [46–48], angiogenesis [49] or even migration and invasion [50] in synovial fibroblasts or endothelial cells. Furthermore, NaHS augmented also phosphorylation of ERK2 and its downstream target MSK2 whereas it had no significant effects on p38 and JNK activation, neither in unstimulated nor in IL-1β stimulated cells.

Fibroblast-like synoviocytes together with macrophages and extracellular matrix form the synovial lining layer which separates the synovial fluid compartment from the synovial sublining region. As demonstrated in previous investigations, RA-FLS spontaneously form a lining layer-like structure in a 3-D micromass culture system showing great similarities with the hyperplastic lining layer in the joints of RA patients [24]. Hence, this culture system represents an attractive *in vitro* model system allowing to explore the effects of stimulating and inhibitory agents. This motivated us to further analyse the cellular consequences of NaHS treatment and its possible effects on the hyperplastic potential of FLS in the 3-D cell culture model.

The data obtained demonstrate that also OA-FLS form a condensed lining layer-like structure and that stimulation with IL-1β resulted in hyperplasia of this lining layer which is consistent with findings made in TNF-α stimulated RA-FLS [24]. Remarkably, the stimulatory effect of IL-1β was completely blocked in cultures treated with NaHS, impressively supporting the results obtained in conventional cultures. Moreover, immunohistochemical analysis demonstrated at the protein level the inhibitory effect of NaHS on MMP-2 expression observed in conventional cultures by RT-PCR, and confirmed phosphorylation of Akt observed by spot and Western blotting. These results suggest that activation of Akt might more likely support cell survival than invasiveness or hyperplasia. The pronounced inhibition of IL-1β-induced cell activation cannot be explained alone by the complex effects on the MAPkinase signal transduction system, but presumably also involves the NF-kB pathway which in FLS is essential for IL-6 regulation [26] and known to be strongly affected by H_2_S [15].

In summary, our study sheds some new light on the role the gasotransmitter H_2_S might play in the pathogenesis of a degenerative joint disease like OA by affecting activation of FLS which are among the cellular key players of this disorder. Thus, on the one hand, the anti-inflammatory properties of H_2_S by its virtue to inhibit IL-1β-induced cytokine production may be beneficial; on the other hand, the effects on signal transduction pathways involved in regulation of cell growth and adhesion are rather complex and difficult to interpret and will be the subject of future investigations. Increasing our understanding of the biological role of H_2_S in synovial inflammation and tissue homoeostasis and elucidating the mode of action of sulphur treatment at the molecular level will form a sound scientific basis for developing of badly needed therapeutic agents for treatment of OA.
